# Introduction to the Molecules Special Edition Entitled ‘*Heparan Sulfate and Heparin: Challenges and Controversies*’: Some Outstanding Questions in Heparan Sulfate and Heparin Research

**DOI:** 10.3390/molecules24071399

**Published:** 2019-04-10

**Authors:** Edwin A Yates, John T Gallagher, Marco Guerrini

**Affiliations:** 1 Department of Biochemistry, University of Liverpool, Crown Street, Liverpool L69 7ZB, UK; 2 University of Manchester and Iduron Ltd, Biohub, Alderley Park, Alderley Edge, Cheshire SK10 4TG, UK; john@iduron.co.uk; 3 Ronzoni Institute for Chemical and Biochemical research, Via G Colombo 81, Milano 20133, Italy

The scope of this article is to provide a brief general introduction to heparan sulfate (HS) and heparin, and attempt to identify some of the central challenges regarding research into the chemistry and biology of glycosaminoglycans (GAGs), some of which are the subject of contributions to the special issue of *Molecules* (published in volume *23*, 2018) entitled ‘*Heparan Sulfate and Heparin: Challenges and Controversies*’. Here, we restrict the definition of chemistry to mean those structural aspects of the subject that relate to biological activity, hence, for example, why the notable progress that has been made in the organic synthesis of these complex molecules will not be discussed. By attempting to enumerate what we see as some of the key challenges to the field, we hope not only to bring them into better focus for our own future research purposes, but also to stimulate productive discussion as how best to tackle them collectively as a research community. 

The GAG research community is, and has to be, extremely diverse to make progress and encompasses biologists, biochemists, chemists, molecular biologists, cell biologists, physicists, pharmacologists, mathematicians and others. This lends the field a fragmented appearance and the first aim is to stimulate researchers to launch further concerted collaborative efforts. An associated aim is to encourage these disparate groups to communicate with one another, discuss the issues from their diverse viewpoints and thereby foster fresh approaches. 

The widespread availability of heparin, which arises as a consequence of its role as a pharmaceutical anticoagulant, compared to the much scarcer HS, has resulted in the application of heparin and its derivatives as proxies for HS. It is therefore worthwhile to begin by examining the extent to which separate definitions of heparin and HS can be justified. One may ask whether these exist as separate biopolymers with distinct biosynthetic control, which could include separate tissue or cell location or, whether they employ the same biosynthetic machinery, but in different ‘geographical’ locations; hence, should they be termed structural variants of essentially the same biopolymer? 

Mammalian HS and heparin are chemically-related GAGs in which a common biosynthetic precursor composed of alternating-4) d-GlcA β(1-4) d-GlcNAc α(1–repeat units is enzymatically-modified by *N*- and *O*-sulphation and uronate epimerization (note that, for clarity, subsequent sequences will be quoted without referring to the linkage positions or anomeric configuration). These modifications give rise to complex polymers that carry out a rich variety of biochemical and physiological activities [1,2]. The key to the wide functional spectrum associated with HS/heparin is their strong propensity to interact with an extensive and diverse range of structural and effector proteins [3,4,5,6]. 

Heparin and HS differ in cellular origin and location. Heparin is synthesised principally as a large (80 kDa) polymer by mast cells, where it is found in intracellular secretory granules in association with mast cell proteases and biogenic amines [7]. In contrast, HS is a ubiquitous component of all cells and tissues being present mainly on cell surfaces and in the extracellular matrix [2,6]. Although they are assembled on different core proteins, it is the extent of polymer-level modifications that enables a molecular distinction to be made between HS and heparin. Heparin is highly sulphated [8,9] and its major disaccharide (~70%) is the trisulphated unit IdoA2S-GlcNS6S [Figure 1] with a degree of polymer sulphation of about 2.4 per disaccharide; only a very minor fraction of disaccharides are unmodified or occur as monosulphated units [10]. There is also some evidence for regulated structure in heparin. The large heparin chain that is produced initially undergoes partial degradation either within the mast cell granules or following secretion. Degradation is carried out by an endoheparanase (hpa1) that specifically cleaves at GlcA- GlcNS(+/−6S) sites to yield fragments of relatively uniform size (~15 kDa) suggesting that these hpaI sites are quite evenly spaced along the chain [11,12]. The 3-0-sulphated high-affinity antithrombin binding sequence appears to be enriched towards the non-reducing ends of the hpa1 cleaved chains [13]. Less sulphated disaccharides tend to be grouped in so-called irregular sections of the heparin chain [14]. There is no evidence to suggest that these irregular regions are spaced in an orderly manner although a sequence of low sulphation resides close to the heparin core protein [15]. In contrast, HS exhibits an ordered polymeric structure that is evident in the organisation of its modified regions [Figure 2] with a molecular structure that distinguishes it clearly not only from heparin, but from all other sulphated GAGs [2,6].

It is well-recognised that HS is less sulphated than heparin with a degree of *N*-sulphation generally in the range of 40–50% of total disaccharides [16]; this relatively low degree of *N*-sulphation, the primary modification in HS biosynthesis, sets a limit on the overall level of *O*-sulphation/GlcA-epimerisation that can be applied to HS, because the specificities of the HS-OSTs and C5-epimerase (see Figure 1) restrict their actions to the vicinity of the *N*-sulphate groups [1,2]. The modifications are not randomly distributed, but show a marked tendency to cluster in extended or block sequences that are quite regularly spaced along the chain and separated by regions largely deficient in sulphate groups ([17], Figure 2)). Two subdomains, the S- (or NS-) domains and the NA/NS- or transition zones (T-zones) can be recognised within the sulphated regions of HS [18]. The S-domains are the more heparin-like regions of HS with a general structure of:

GlcA-GlcNS-[IdoA2S-GlcNS (+/-6S)]_n_-GlcA/IdoA-GlcNAc

in which the number of internal IdoA2S-GlcNS disaccharide units, n, ranges from 1 to 7. 

S-domains can be excised by heparinase III (Hep III) which acts mainly on GlcNAc/GlcNS-GlcA linkages; the presence of IdoA2S inhibits Hep III and this enables the preparation of S-domains for sequence analysis and investigation of their biological properties [19]. The NA/NS regions flank the S-domains and have a relatively low level of sulphation [20]; in such sequences (general structure GlcA/IdoA-GlcNAc-GlcA-GlcNS) either or both amino sugars can be sulphated at C6 and in some species of HS the level of 6-sulphation exceeds that of the S-domains [6]. These regions make up about 25% of a typical HS chain and are likely to be functionally significant. It is conceivable that novel conformations with particular recognition properties may reside at the NA/NS and S-domain interface. Heparin on the other hand is largely devoid of NA/NS regions. The ‘sulphation patterns’ in the modified regions of HS vary between different cells and tissues [15,21,22]; the variations seem to be determined genetically and may conceivably represent some form of signature on the cell surface that is recognised by extrinsic regulatory proteins. The more sparing use of sulphation in HS is likely to confer greater discrimination in protein binding than that observed with heparin. 

An additional notable feature of the HS chain, not seen in heparin, is that the proximal region near to the heparan sulfate proteoglycan (HSPG) core protein is composed of an *N*-acetylated (NA) segment about 10 disaccharides in length that lacks any enzymatic transformation [23]. It is unclear how this region consistently escapes modification, but hints at steric interference. Intriguingly, it now appears that S-domains, often highly sulphated, are common at the periphery of the HS chain remote from the protein core. These ‘exposed’ and probably more accessible S-domains display enhanced reactivity in fibroblast growth factor (FGF)-binding and mitogenic assays [24]. 

There are sources of mammalian ‘HS’ that do not conform strictly to the design shown in Figure 2. This is the *N*-sulphated polymer synthesised by rat liver. The liver HS is a short chain (~ 22 kDa compared with ~ 40 kDa for the common forms of HS) with a highly asymmetric organisation in which a proximal, non-sulphated core sequence is linked to three highly-sulphated S-domains positioned towards the distal end [25]. The S-domains are separated by short Hep III- sensitive NA and NA/NS regions (Figure 2). This structure clearly has both HS and heparin-like characteristics. A second example concerns ‘heparin-like’ heparan sulphates that have been isolated from the growth plate and articular cartilage of young rabbits, which contains high levels of sulphation, and unexpectedly, also the pentasaccharide sequence responsible for antithrombin binding [26]. It is interesting that cultured glial cell progenitors synthesise an *N*-sulphated GAG on the cell surface that clearly resembles prototypical heparin more closely than HS [27]. In contrast, differentiated glial subpopulations (oligodendrocytes and astrocytes) synthesise typical HS structures but with distinctive fine structural features for each cell type [27]. It is not known to what extent these forms of HS are the consequence of cell culture conditions and passage number. Heparin can be considered a novel differentiation marker in the glial lineage. Again, one may ask whether this is a unique feature, or whether other immature cell populations produce heparin at specific stages of development.

Assessing the evidence available so far, it seems reasonable to conclude that HS and heparin are distinctive polymers in which the highly-sulphated and relatively uniform heparin chain contrast with the domain organisation and cell-specific sulphation patterning of the most common appearance of HS. The two do not represent a continuum of structures and are likely to be the result of different modes of biosynthesis; however, rat liver HS partially blurs this distinction and as more data become available on HS from other cell types a range on intermediate forms may emerge that fill the sulphation gap between prototypical HS and heparin. 

One further point is that the data so far discussed refer to mammalian HS and heparin. Of the various model organisms used in developmental studies, only *Drosophila* HS has been examined in detail. The *Drosophila* HS is a relatively short (~30 disaccharides in length) two domain structure with HS- and heparin-like features [28]. It consists of an internal extended acetylated (NA) sequence, as found in mammalian HS, connected to a long S-domain similar to, though less sulphated than, heparin. Although many variations of the *Drosophila* ‘prototype’ are likely to exist in the *insecta* class [29], it seems that in the course of mammalian evolution a complex HS polymer emerged with extension of chain length, the appearance of segregated S-domains, but with the retention of the core NA sequence. Heparin of similar structure to mammalian heparin is present in the *mollusca,* which predate the *insecta* in the evolutionary tree [30]. The persistence of heparin through a long period of evolution suggests that it is essential for adaption to environmental change. Indeed, it has been suggested that the origin of GAGs, including HS, coincides with the onset of multicellular life and, in particular, neuronal connectivity [31].

The debate around what constitutes heparin or HS also highlights the question of how the two biopolymers—or, indeed, any GAG biopolymers—should best be compared? The straightforward answer of a comparison of sequence is itself fraught with technological challenges but, even if this could be achieved, extensive evidence shows that sequence alone—that is, without further interpretation—will be unlikely to be able to identify, for instance, those sequences that could bind a given protein. The extensive redundancy exhibited between sequences of heparin and HS-derived oligosaccharides demonstrates that whatever the required features for binding and activity are (and note that the first of these does not necessarily provide sufficient condition for the second), they reside in a combination of conformation, the geometric presentation and orientation of charged groups, their interactions with counter ions and dynamic aspects such as flexibility [32]. The action of HS on proteins can alter protein function by several means, which include stabilization, altering protein conformation, causing oligomerization, altering presentation of a protein to its receptor, or through the formation of morphogen gradients [33].

The underlying mechanisms of HS and heparin biosynthesis were uncovered over several decades, establishing the basic order of enzymatic action. Accumulated data from many detailed studies [34] permitted a biosynthetic scheme at the level of disaccharides to be suggested, which proposed two branches, distinguished by the efficacy of a single enzyme, epimerase, on two substrates—one comprising GlcA-GlcNS and the other GlcA-GlcNAc to generate the subsequent IdoA containing disaccharide units [35]. Subsequent findings in relation to 3-O-sulfotransferase activity, nominally the final enzymatic step (excluding subsequent *sulfatase* activity), were consistent with this hypothesis [36]. Nevertheless, the full regulation of HS biosynthesis is not at all well-understood and, while the above disaccharide-based scheme may provide one step, it is incomplete, not least because it does not take into account the effect of adjacent structures—i.e., it does not deal with oligosaccharides. The advent of the biosynthesis of defined oligosaccarides of Hp or HS [37] offers an important opportunity for studying the influence of sequence on enzyme action, with the limitation that such experiments currently usually occur in solution, rather than in cellular compartments; thus, any additional control mechanisms cannot be observed.

A related feature of biosynthesis that remains refractory is the control of domain synthesis. A key question relates to which enzymes combine to form multi-enzyme complexes during biosynthesis and how they do so. This is one area in which the technological challenges are formidable, but future progress through tracking enzyme expression through the biosynthetic process is anticipated. A further complication in HS structure-function elucidation arises from the observation that different cation forms of heparin, modified heparins (serving again as proxies for HS) exhibit distinct conformational and dynamic properties and that these give rise, in several cases, to distinct biological responses [38,39]; nevertheless, a degree of redundancy has been repeatedly observed, when varied Hp or HS structures have been tested for biological activity. Parallel studies that employ cationic versions of HS, rather than heparin, are awaited. Coincidentally, the high degree of conformational and charge resemblance between saccharides with distinct sequences [40] may explain why separating mixtures of oligosaccharides from these biopolymers that are of similar length and charge is so challenging.

If HS follows a similar trend to heparin, an interesting possibility follows. Either the selectivity of the polysaccharide in different cation forms is very relaxed and the polysaccharide is simply subject to whatever ions are present at a given location combined with the relative affinities between cation and structure, or the biosynthesis of the HS is linked to the regulation of cations, or of particular cations. In this regard, a surprising finding [41] was that heparin, when exposed to calcium ions at both lower and elevated physiologically relevant temperatures (280 and 305 K) adopted seemingly conserved conformations, as judged by their indistinguishable ^1^H-NMR spectra. This is distinct from the sodium ion form, which undergoes conformational change between these temperatures and the result suggests a possible link between HS structure (i.e., biosynthesis) and calcium regulation. Given the role of Hp in response to tissue damage and inflammation, coupled with the harmful effects of calcium ions, this could serve the role of capturing toxic calcium ions following tissue damage across a range of temperatures, while maintaining regulation of protein networks (since the heparin conformation is seemingly unchanged). It is currently not known what the effects of distinct Hp or HS structures are, nor the influence that other ions, especially other divalent ions such as magnesium, zinc or copper have in this regard. 

This special issue consists of eight papers, comprising both reviews and original research articles and these are summarised briefly below. Akhtar et al., reviewed the application of low molecular weight heparin in micro- and nanoparticles and concluded that further improvements in the efficiency of the delivery systems will be required if the benefits of these materials, especially in medical applications, are to be fully realised [42]. For many years, the primary method for elucidating the detailed solution conformation of HS and heparin-derived oligosaccharides has been nuclear magnetic resonance spectroscopy (NMR) and, in particular, the interpretation of three bond proton-proton (^3^J_H-H_) coupling constants. Hricovini and Hricovini reviewed the state of the art, including the use of density functional theory (DFT), highlighting the importance of employing the appropriate quantum chemical calculations that need to be able to account for the unexpected contribution that oxygen lone pairs make to Fermi-contact contributions in these molecules [43]. In more biologically oriented research, the article by Veraldi et al., reported, for the first time, differences in the detailed structure of HS that has been derived from osteochondromas, chondrosarcomas and healthy cartilage [44]. This work opens up a new area for investigation, which has been little studied and highlights the importance of detailed structural characterization of these materials. Ponert et al., reported their work on an important phenomenon in cancer biology, the ability of platelets to activate the epithelial-mesenchymal transition and confer stem-like properties onto tumour cells with increased drug resistance and enhanced motility. Both unfractionated and low molecular weight heparin reduced this platelet-induced transition and highlights the potential of heparin in oncological applications [45]. In a further report concerning the activity of platelets in cancer, Gockel et al., showed that low molecular weight heparin has the ability to moderate the platelet activation by two routes; through both coagulation dependent and independent mechanisms [46]. Continuing the cancer theme, Hellec et al., described the role of one of the sulfotransferase enzymes, HS3ST3B, responsible for the addition of 3-O-sulfate groups to glucosamine residues in HS, in enhancing tumour growth, which the authors showed is dependent on the expression of neuropilin-1 [47]. Extending the earlier discussion of the ability of heparin and its derivatives to mimic HS and encompassing other classes of mimics, Lanzi and Cassinelli reviewed the diverse roles that these mimics serve [48]. The key finding that emerged was that multiple targets can be influenced by HS mimetics, including heparanase, selectins, growth factors and the immune system. Regarding one of these activities, the ability to inhibit heparanase activity, the review by Chhabra and Ferro highlighted an important technical point. This relates to the difficulty of comparing published results that are derived from diverse assay systems [49]. The authors called for a standardised approach to these measurements, requiring reliable and reproducible assays, which is an important point to allow additional progress to be made in this field. Another pertinent observation, this time relating to GAG-protein binding and its relevance to drug development, was made by Boittier et al., [50] who compared GAG binding sites on antithrombin III, heparanase and in chemokines (sub-families CCL and CXCL) between species using informatics and modelling approaches. Interestingly, they found that the choice of species for an animal model could be important, since in some cases, there are differences in GAG binding sites that lead to distinct architectures for the GAG-protein complex. The authors recommend that these inter-species differences should be incorporated into the decision making process when selecting model systems for drug development.

The ability of HS and its mimetics, including heparin and its derivatives, to interact with multiple systems, in some cases entire signalling systems, has been remarked on in several guises in the past and this is emerging as a key property of both HS and of molecules that are able to mimic it. Early efforts to assess the significance of these networks have concentrated on a related family of signalling proteins, the fibroblast growth factor (FGF) family [51,52] through their interactions with FGF-receptors (FGFRs) [53], although several other families of proteins including those involved in inflammation [54] seem to merit further study in this regard.

## Figures and Tables

**Figure 1:**
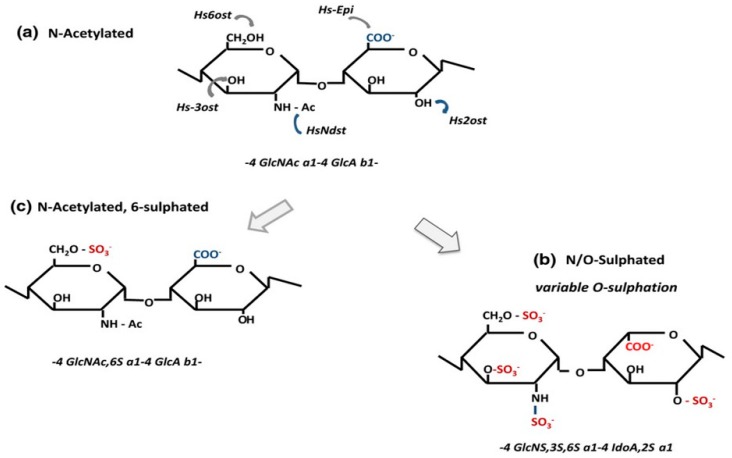
Enzymatic Modifications in the Biosynthesis of Heparin and Heparan Sulphate. Heparin and heparan sulphate (HS) are first synthesised as non-sulphated polymers (GlcNAc-GlcA repeat units) that are modified to different degrees by a combination of *N*- and *O*-sulphation and epimerisation. The initial modification step carried out by the Ndst enzymes is the conversion of GlcNAc to GlcNS; in heparin, about 90% of GlcNAc residues are converted to the *N*-sulphated derivative whereas in HS the level of conversion is normally in the range of 40 to 50%. In a mature heparin polymer, the main disaccharide is a trisulphated unit (GlcNS6S-IdoA2S), whereas in HS the sulphated and epimerised disaccharides occur in clusters and with a low frequency (normally below 10%) of trisulphated units. In both polymers, 3-*O*-sulphation, though rare, is a key functional group in the high-affinity antithrombin binding sequence, but can also be found in non-anticoagulant sequences. Key to enzymes: Hs*Ndst*: *N*-deacetylase/*N*-sulphotransferase, Hs*epi*: C5-epimerase*, Hs2ost*: 2-*O*-sulphotransferase, *Hs6ost*: 6-*O*-sulphotransferase, *Hs3ost* 3-*O*-sulphotransferase. This diagram was originally published in [6].

**Figure 2:**
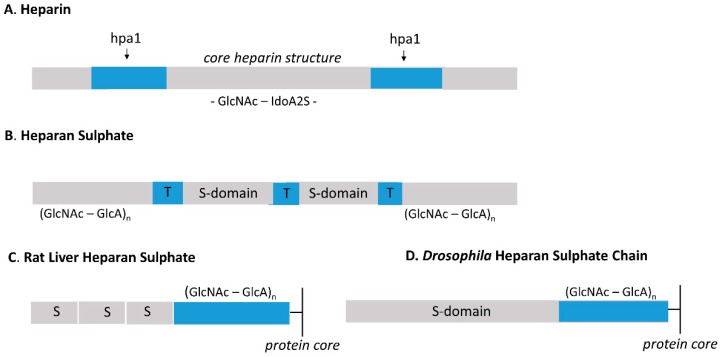
Differences in molecular design between characteristic regions of heparin (**A**) and heparan sulphate (**B**). The structure of the highly sulphated heparin is dominated by the trisulphated GlcNS6S—IdoA2S unit; however, there are less sulphated sequences that can be cleaved by an endoheparanase, hpa1, distributed throughout the chain at approximately 15 kDa intervals. The molecular design of HS is quite distinct from that of heparin. The sulphated regions composed of S- and NA/NS-domains (or T-zones) are arranged in a regular manner along the glycosaminoglycan (GAG) chain separated by extensive areas that lack any enzymatic modification (NA domains). A long NA sequence of nine to 10 disaccharides is present in the inner region of HS proximal to the protein core (shown in (**C**,**D**)), depicting, respectively, the asymmetric rat liver HS and the two-domain *Drosophila* HS). A comparable sequence is not present in heparin. The “composite regions” of sulphation in HS illustrated above are ~ 7 kDa in size and can be excised by K5- heparan lyase that acts specifically on NA sequences. This enzyme is inhibited by the presence of the *N*-sulphate group. Hpa1 susceptible sequences are present in HS probably at the junction of NA/NS and S-domains.

## References

[1] Kreuger J., Kjellen L. (2012). Heparan sulfate biosynthesis—Regulation and variability. J. Histochem. Cytochem..

[2] Li J.-P., Kusche-Gullberg M. (2016). Heparan sulfate: Biosynthesis, structure, and function. Int. Rev. Cell Mol. Biol..

[3] Casu B., Lindahl U. (2001). Structure and biological interactions of heparin and heparan sulfate. Adv. Carbohydr. Chem. Biochem..

[4] Skidmore M.A., Guimond S.E., Rudd T.R., Fernig D.G., Turnbull J.E., Yates E.A. (2008). The activities of heparan sulfate and its analogue heparin are dictated by biosynthesis, sequence, and conformation. Connect. Tissue Res..

[5] Forster M., Mulloy B. (2006). Computational approaches to the identification of heparin binding sites on the surfaces of proteins. Biochem. Soc. Trans..

[6] Gallagher J. (2015). Fell-Muir Lecture: Heparan sulphate and the art of cell regulation: A polymer chain conducts the protein orchestra. Int. J. Exp. Path..

[7] Ronnberg E., Melo F., Pejler G. (2012). Mast cell proteoglycans. J. Histochem. Cytochem..

[8] Mulloy B., Lever R., Mulloy B., Page C.P. (2012). Structure and physicochemical characterization of heparin. Heparin: A Century of Progress.

[9] Stringer S., Kandola B., Pye D., Gallagher J. (2003). Heparin sequencing. Glycobiology.

[10] Conrad H.E. (1997). Heparin Binding Proteins.

[11] Wang A., Sankaranarayanan N.V., Yanagishita M., Templeton D.M., Desai U.R., Sugahara K., Wang C.P., Hascall V.C. (2015). Heparin interaction with a receptor on hyperglycemic dividing cells prevents intracellular hyaluronan synthesis and autophagy responses in models of type 1 diabetes. Matrix Biol..

[12] Gong F., Jemth P., Escobar-Galvis M.L., Vlodavsky I., Horner A., Lindahl U., Li J. (2003). Processing of macromolecular heparin by heparinase. J. Biol. Chem..

[13] Radoff S., Danifeshefsky I. (1984). Location on heparin of the oligosaccharide section essential for anticoagulant activity. J. Biol. Chem..

[14] Yamada S., Yamane Y., Tsuda H., Yoshida K., Sugahara K. (1998). A major common trisulfated hexasaccharide core sequence, hexuronic acid(2-sulfate)-glucosamine(N-sulfate)-iduronic acid-N-acetylglucosamine-glucuronic acid-glucosamine(N-sulfate), isolated from the low sulfated irregular region of porcine intestinal heparin. J. Biol. Chem..

[15] Sugahara K., Tsuda H., Yoshida K., de Beer T., Vliegenthart J. (1995). Structure determination of the octa- and decasaccharide sequences isolated from the carbohydrate-protein linkage region of porcine intestinal heparin. J. Biol. Chem..

[16] Gallagher J., Walker A. (1985). Molecular distinctions between heparan sulphate and heparin. Analysis of sulphation patterns indicates that heparan sulphate and heparin are separate families of N-sulphated polysaccharides. Biochem. J..

[17] Turnbull J., Gallagher J. (1991). Distribution of iduronate 2-sulphate residues in heparan sulphate. Evidence for an ordered polymeric structure. Biochem. J..

[18] Murphy K., Merry C., Lyon M., Thompson J.E., Roberts I.S., Gallagher J.T. (2004). A new model for the domain structure of heparan sulfate based on the novel specificity of K5 lyase. J. Biol. Chem..

[19] Merry C., Lyon M., Deakin J., Hopwood J., Gallagher J.T. (1999). Highly sensitive sequencing of the sulphated domains of heparan sulphate. J. Biol. Chem..

[20] Sanderson P.N., Huckerby T.N., Nieduszynski I.A. (1984). Very-high-field n.m.r. studies of bovine lung heparan sulphate tetrasaccharides produced by nitrous acid deaminative cleavage. Determination of saccharide sequence, uronate composition and degrees of sulphation. Biochem. J..

[21] Shi X., Zaia J. (2009). Organ-specific heparan sulphate structural phenotypes. J. Biol. Chem..

[22] Ledin J., Staatz W., Li J.P., Gotte M., Selleck S., Kjellen L., Spillman D. (2004). Heparan sulfate structure in mice with genetically modified heparan sulfate production. J. Biol. Chem..

[23] Lyon M., Steward W.P., Hampson I., Gallagher J.T. (1987). Identification of an extended N-acetylated sequence adjacent to the protein-linkage region of heparan sulphate. Biochem. J..

[24] Naimy H., Buczek-Thomas J.A., Nugent M., Leymarie N., Zaia J. (2011). Highly sulfated nonreducing end-derived heparan sulfate domains bind fibroblast growth factor-2 with high affinity and are enriched in biologically active fractions. J. Biol. Chem..

[25] Lyon M., Deakin J., Gallagher J.T. (1994). Liver heparan sulphate structure: a novel molecular design. J. Biol. Chem..

[26] Parra A., Veraldi N., Locatelli M., Fini M., Martini L., Torri G., Sangiorgi L., Bisio A. (2012). Heparin-like heparin sulfate from rabbit cartilage. Glycobiology.

[27] Stringer S., Mayer-Proschel M., Kalyani A., Rao M., Gallagher J.T. (1999). Heparin is a unique marker of progenitors in the glial cell lineage. J. Biol. Chem..

[28] Kusche-Gullberg M., Nybakken K., Perrimon N., Lindahl U. (2012). Drosophila heparan sulfate, a novel design. J. Biol. Chem..

[29] Rogerson E., Pelletier J., Acosta-Serrano A., Rose C., Taylor S., Guimond S., Lima M., Skidmore M., Yates E. (2018). Variations in the peritrophic matrix composition of heparin sulfate from the tsetse fly, *Glossina morsitans morsitans*. Pathogens.

[30] Dietrich C.P., de Paiva J., Moraes C.T., Takahashi H.T., Porcionatto M.A., Nader H.B. (1985). Isolation and characterization of a heparin with high anticoagulant activity from Anomalocardia brasiliana. Biochim. Biophys. Acta.

[31] Van Vactor D., Wall D.P., Johnson K.G. (2006). Heparan sulfate proteoglycans and the emergence of neuronal connectivity. Curr. Opin. Neurobiol..

[32] Meneghetti M.C., Hughes A.J., Rudd T.R., Nader H.B., Powell A.K., Yates E.A., Lima M.A. (2015). Heparan sulfate and heparin interactions with proteins. J. R. Soc. Interface.

[33] Ori A., Wilkinson M.C., Fernig D.G. (2008). The hepanosome and regulation of cell functions: Structures, functions and challenges. Front. Biosci..

[34] Mizumoto M., Kitagawa H., Sugahara K., Garg H.G., Linhardt R.J., Hales C.A. (2005). Biosynthesis of heparin and heparin sulfate. Chemistry and Biology of Heparin and Heparan Sulfate.

[35] Rudd T.R., Yates E.A. (2012). A highly efficient tree structure for the biosynthesis of heparin sulfate accounts for the commonly observed disaccharides and suggests a mechanism for domain synthesis. Mol. Biosyst..

[36] Meneghetti M.C.Z., Ferreira T.G., Tashima A.K., Chavante S.F., Yates E.A., Liu J., Nader H.B., Lima M.A. (2017). Insights into the role of 3-O-sulfotransferases in heparan sulfate biosynthesis. Org. Biomol. Chem..

[37] Zhang X., Pagadala V., Jester H.M., Lim A.M., Pham T.Q., Goulas A.M.P., Liu J., Linhardt R.J. (2017). Chemoenzymatic synthesis of heparan sulfate and heparin oligosaccharides: paving the way to a diverse library for glycobiologists. Chem. Sci..

[38] Rudd T.R., Guimond S.E., Skidmore M.A., Duchesne L., Guerrini M., Torri G., Cosentino C., Brown A., Clarke D.T., Turnbull J.E. (2007). Influence of substitution pattern and cation binding on conformation and activity in heparin derivatives. Glycobiology.

[39] Guimond S.E., Rudd T.R., Skidmore M.A., Ori A., Gaudesi D., Cosentino C., Guerrini M., Edge R., Collison D., McInnes E. (2009). Cations modulate polysaccharide structure to determine FGF-FGFR signaling: A comparison of signaling and inhibitory polysaccharide interactions with FGF-1 in solution. Biochemistry.

[40] Rudd T.R., Yates E.A. (2010). Conformational degeneracy restricts the effective information content of heparan sulfate. Mol. Biosyst..

[41] Hughes A., Meneghetti M., Hung T.-Y., Huang S.-C., Elli S., Guerrini M., Rudd T., Lima M., Yates E. (2017). Investigating the relationship between temperature, conformation and Ca binding in heparin model oligosaccharides. Carbohydr. Res..

[42] Akhtar F., Wan X., Wu G., Kesse S., Wang S., He S. (2018). Low molecular weight heparins: Reduced size particulate systems for improved therapeutic outcomes. Molecules.

[43] Hricovini M., Hricovini M. (2018). Solution conformation of heparin tetrasaccharide. DFT analysis of structure and spin-spin coupling constants. Molecules.

[44] Veraldi N., Parra A., Urso E., Cosentino C., Locatelli M., Corsini S., Pedrini E., Naggi A., Bisio A., Sangiorgi L. (2018). Structural features of heparin sulfate from multiple osteochondromas and chondrosarcomas. Molecules.

[45] Ponert J.M., Gockel L.M., Henze S., Schlesinger M. (2018). Unfractionated and low molecular weight heparin reduce platelet induced endothelial-mesenchymal transition in pancreatic and prostate cancer cells. Molecules.

[46] Gockel L.M., Ponert J.M., Schwarz S., Schlesinger M., Bendas G. (2018). The low molecular weight heparin tinzaparin attenuates platelet activation in terms of metastatic niche formation. Molecules.

[47] Hellec C., Diawara M., Carpentier M., Denys A., Allain F. (2018). The pro-tumoral activity of heparan sulfate 3-O-sulfotransferase 3B (HS3ST3B) in breast cancer MDA-MB-231 cells is dependent on the expression of neuropilin-1. Molecules.

[48] Lanzi C., Cassinelli G. (2018). Heparan sulfate mimetics in cancer therapy. Molecules.

[49] Chhabra M., Ferro V. (2018). The development of assays for heparanase enzymatic activity: Towards a gold standard. Molecules.

[50] Boittier E.D., Gandi N.S., Ferro V., Coombe D.R. (2019). Cross-species analysis of GAG binding proteins reveals some animal models are ‘more equal than others’. Molecules.

[51] Xu R., Ori A., Rudd T.R., Uniewicz K.A., Ahmed Y.A., Guimond S.E., Skidmore M.A., Siligardi G., Yates E.A., Fernig D.G. (2012). Diversification of the structural determinants of fibroblast growth factor-heparin interactions: Implications for binding specificity. J. Biol. Chem..

[52] Li Y., Sun C., Yates E.A., Jiang C., Wilkinson M.C., Fernig D.G. (2016). Heparin binding preference and structures in the fibroblast growth factor family parallel their evolutionary diversification. Open Biol..

[53] Xu R., Rudd T.R., Hughes A.J., Siligardi G., Fernig D.G., Yates E.A. (2013). Analysis of the fibroblast growth factor receptor (FGFR) signaling network with heparin as coreceptor: Evidence for the expansion of the core FGFR signalling network. FEBS J..

[54] Veraldi N., Hughes A.J., Rudd T.R., Thomas H.B., Edwards S.W., Hadfield L., Skidmore M.A., Siligardi G., Cosentino C., Shute J.K. (2015). Heparin derivatives for the targeting of multiple activities in the inflammatory response. Carbohydr. Polym..

